# Multivariable Prognostic Models for Age‐Related Macular Degeneration: A Systematic Review

**DOI:** 10.1155/joph/9231744

**Published:** 2026-09-18

**Authors:** Rumaisa Aljied, Simran Saggu, Varun Chaudhary, Marie Pigeyre, Lauren E. Griffith, Parminder Raina

**Affiliations:** ^1^ Department of Health Research Methods, Evidence, and Impact, Faculty of Health Science, McMaster University, Hamilton, Ontario, Canada, mcmaster.ca; ^2^ McMaster Institute for Research on Aging, McMaster University, Hamilton, Ontario, Canada, mcmaster.ca; ^3^ Department of Ophthalmology, McMaster University, Hamilton, Ontario, Canada, mcmaster.ca; ^4^ Department of Medicine, McMaster University, Hamilton, Ontario, Canada, mcmaster.ca

**Keywords:** age-related macular degeneration, disease progression, geographic atrophy, machine learning, neovascular AMD, optical coherence tomography, prognostic prediction models

## Abstract

**Background:**

Prognostic prediction models for age‐related macular degeneration (AMD) are proliferating, driven by machine learning applied to retinal imaging. Although the methodological limitations of clinical prediction models are well recognized in general, AMD‐specific multivariable prognostic models, as distinct from diagnostic or image‐classification models, have not been appraised against contemporary prediction‐model standards, with integrated assessment of outcome definitions, prediction horizons, calibration, validation design, and risk of bias.

**Objective:**

To systematically identify and critically appraise multivariable prognostic models predicting progression from non‐late to late AMD and to determine whether quantitative synthesis was appropriate, with emphasis on model design, predictors, performance reporting, validation, and risk of bias.

**Methods:**

We searched PubMed/MEDLINE, and Embase from inception to April 2022, updated to December 2024, for studies developing, validating, or evaluating multivariable prognostic models for progression to late AMD. We extracted study population, outcomes, prediction horizons, predictors, modeling methods, performance measures, and validation approach and assessed risk of bias with PROBAST. Given substantial heterogeneity, findings were synthesized narratively in accordance with a prespecified conditional analysis plan (PROSPERO CRD42022323522).

**Results:**

Twenty studies were included. Models were derived from a small number of recurring cohorts, notably AREDS and HARBOR, of predominantly European ancestry; this limited population diversity was one of several constraints, alongside heterogeneous outcome definitions, infrequent calibration, and uncommon external validation. Approaches ranged from regression‐based risk scores to deep learning applied to fundus photography, optical coherence tomography, or multimodal data. Because outcome definitions varied (composite late AMD, neovascular or exudative conversion, geographic atrophy or other atrophic endpoints, and treatment‐initiation proxies), the observed AUC/c‐statistic range (∼0.65–0.97) reflects differing prediction tasks rather than directly comparable performance. Calibration was addressed in six studies (30%), and fewer still met our definition of quantitative calibration. Imaging features and age were the most consistently retained predictors, and measures beyond discrimination were rare. Overfitting could not be excluded in several studies, as event counts, candidate‐predictor numbers, use of shrinkage, and internal‐validation procedures were incompletely reported. Ten of 20 studies were at low overall risk of bias, but calibration and external validation were uncommon across the evidence base as a whole.

**Conclusions:**

Prognostic models for AMD progression are methodologically diverse but are drawn from few, homogeneous cohorts, with limited calibration and external validation, features that constrain interpretation of reported performance and transportability to routine, more diverse practice. Progress will depend less on new algorithms than on staged, feasible validation: prespecified outcomes and horizons, robust internal validation, routine calibration, and temporal or geographic validation with recalibration before clinical use. Until then, existing models should be regarded as exploratory rather than clinically actionable.

## 1. Introduction

Age‐related macular degeneration (AMD) is a leading cause of irreversible vision loss worldwide and represents a growing health burden in aging populations [1, 2]. Although AMD is commonly categorized into early, intermediate, and late stages, progression from nonlate to late disease varies between individuals [3, 4]. Some patients remain stable for years while others progress rapidly to geographic atrophy or neovascular AMD, outcomes associated with profound and often irreversible visual impairment. This heterogeneity limits the clinical utility of stage‐based classification and creates uncertainty in monitoring and management, particularly when progression risk is misestimated [2, 3].

Clinical decisions in AMD depend on anticipating progression [5]. Follow‐up frequency, imaging intensity, referral to retinal specialists, patient counseling, and the timing of intervention, particularly antivascular endothelial growth factor (anti‐VEGF) therapy, are all influenced by perceived risk of progression [6, 7]. Overestimation of risk will expose patients to unnecessary visits and testing, while underestimation delays detection of treatable disease, with measurable consequences for visual outcomes [7]. In this context, individualized prognostic risk estimates are increasingly viewed as a potential tool to support more targeted, efficient, and equitable care [8].

Multivariable prognostic prediction models aim to estimate the probability of a future outcome given a set of risk factors that can be demographic, clinical, genetic, or imaging in nature [9, 10]. For AMD, these models have evolved from regression‐based risk scores derived from longitudinal cohorts to machine learning and deep learning approaches incorporating fundus photography, optical coherence tomography (OCT), or multimodal data [11–14]. Many models report strong discrimination in development datasets, often with area under the curve (AUC) or concordance (c) statistics exceeding 0.80 [11, 15]. However, discrimination alone does not establish clinical usefulness [16]. For prognostic models intended to guide surveillance or intervention, accurate absolute risk estimation, calibration across risk strata, and performance stability in new populations are essential [16, 17].

Despite rapid methodological advances, the clinical readiness of existing AMD prognostic models remains unclear. Many models are developed in a small number of recurring cohorts or trials [18, 19], often with limited racial and ethnic diversity [20], and rely primarily on internal validation. Reporting of calibration, handling of missing data, and assessment of overfitting are inconsistent [21, 22]. External validation is uncommon, and few studies explicitly address applicability to clinical settings beyond the development population [14]. These limitations raise concerns about risk of bias, transportability, and the potential for harm if models are implemented without adequate validation or recalibration [14, 16, 23].

Several prior reviews have examined deep learning models in AMD, primarily focusing on diagnostic accuracy or automated image classification [21, 22, 24, 25]. To date, no systematic review has evaluated multivariable prognostic models for progression to late AMD with explicit, integrated assessment of outcome definitions, prediction horizons, calibration, validation design, analytic unit, and risk of bias; general appraisals of prediction‐model quality, in turn, have not been disease‐specific. We therefore address this gap directly.

We conducted a systematic review to identify and critically appraise multivariable prognostic prediction models for progression from nonlate to late AMD. Guided by the transparent reporting of a multivariable prediction model for individual prognosis or diagnosis (TRIPOD) [26] and the prediction model risk of bias assessment tool (PROBAST) [27] for prediction‐model reviews, we examined model characteristics, predictor domains, outcomes and time horizons, performance metrics, validation approaches, and risk of bias. Our objective was not to recommend specific models for clinical use, but to assess the current state of the evidence, identify methodological strengths and limitations, and outline priorities for future research aimed at developing reliable, generalizable prognostic tools for AMD care.

## 2. Methods

This systematic review was conducted in accordance with the Preferred Reporting Items for Systematic Reviews and Meta‐Analyses (PRISMA) guidelines [28] and was registered in PROSPERO (CRD42022323522).

### 2.1. Eligibility Criteria

For the purposes of this review, a prognostic prediction model was defined a priori as any multivariable model that estimated the probability or risk of future progression from nonlate to late AMD, including geographic atrophy or neovascular AMD, over a specified prediction horizon, conditional on predictors measured at baseline. Nonlate AMD was defined as the absence of late AMD at baseline and encompassed several starting states: no AMD (where eligible), early AMD, intermediate AMD, or an at‐risk fellow eye without late AMD. These are clinically distinct baseline‐risk groups; we did not treat them as a single homogeneous population, and baseline state is reported per study in Table 1. Models predicting short‐term conversion, imminent exudation, or initiation of treatment were included if the prediction target represented a future event occurring after baseline assessment rather than concurrent disease status. Prediction horizon length was treated as a model characteristic rather than an inclusion criterion. Models were excluded if they were purely diagnostic or classificatory in nature, including those that identified current disease severity, baseline AMD stage, or concurrent exudation without predicting a future outcome beyond the index assessment. Eligible studies were those that developed, validated, updated, or evaluated a multivariable prognostic prediction model for AMD progression, reported at least one measure of model performance, included human participants diagnosed with AMD, and provided sufficient methodological detail to permit extraction of model characteristics and performance metrics. Preprints were eligible when they met all other inclusion criteria. Studies were excluded if they focused solely on diagnostic classification, reported only individual risk factors without model development, were review articles or conference abstracts, or lacked sufficient information on model development or validation. Only studies published in English were considered in this review.

**TABLE 1:** Baseline demographic and clinical characteristics of study populations included in models for predicting age‐related macular degeneration.

Study id	Population	Recruitment period	Inclusion criteria	Exclusion criteria	Age, mean, median (yr)	Sex (% females)	Ethnicity	SES	Average follow‐up (yr)	Number of events (outcome)
Chiu et al. [12]	AREDS: US multicentre clinical cohort recruited at retinal specialty clinics; BMES validation: Australian population‐based cohort	AREDS enrollment Nov 1992–Jan 1998 (follow‐up through 2001); BMES baseline 1992–1994	AREDS: age 55–80; VA 20/32 or better in ≥ 1 eye; lens/vitreous clear enough for quality retinal photographs; ≥ 1 eye free of diseases complicating AMD assessment; no prior ocular surgery except cataract surgery; unilateral photocoagulation for AMD allowed	AREDS: illnesses/disorders making long‐term follow‐up or protocol compliance unlikely/difficult	AREDS mean 68.8 years	AREDS ≈ 56%; BMES 57%	AREDS 97% White	Education reported (proxy)	AREDS mean 6.3 years; BMES 10.5 years	1185 (AREDS eyes progressed to advanced AMD); 69 (BMES eyes progressed)
Buitendijk et al. [29]	Population‐based cohorts from the Netherlands (Rotterdam Study), Wisconsin USA (Beaver Dam Eye Study), and Australia (Blue Mountains Eye Study)	Rotterdam Study baseline 1990–1993; Beaver Dam baseline 1988–1990; Blue Mountains baseline 1992–1994	Genotype data available; gradable baseline fundus photographs; ≥ 1 follow‐up eye examination	Late AMD at baseline	Mean ages: RS 67.6; BDES 60.2; BMES 64.0	RS 57.6%; BDES 57.5%; BMES 57.2%	RS ≈ 98% White; BDES ≈ 99% White (BMES predominantly White)	NR	Median 11.1 years	363 incident late AMD cases
Yan et al. [30]	AREDS participants (US multicentre cohort; eye‐level analysis)	AREDS enrollment Nov 1992–Jan 1998 (follow‐up extended beyond the trial phase)	Age 55–80; Caucasian/White participants; ≥ 1 eye free of late AMD at baseline; ≥ 1 follow‐up visit; fundus images and genotype available	Illness/condition making long‐term follow‐up unlikely; lost to follow‐up	Mean 68.8 years (SD 5.0)	≈ 56% (*N* = 750)	White/Caucasian (analysis restricted)	NR (no income/education reported)	Mean 10.3 years (SD 1.6)	2678 eyes at risk at baseline; progression to late AMD reported by baseline severity strata (4%, 50%, 92% across strata)
Wu et al. [31]	LEAD trial participants with bilateral large drusen without late AMD at baseline (Australia and Northern Ireland)	July 26, 2012–April 30, 2015	Age ≥ 50; ≥ 1 large drusen (> 125 μm) within 1500 μm of fovea in both eyes; BCVA 20/40 or better in both eyes; ≥ 1 follow‐up visit	Late AMD at baseline; other ocular/systemic/neurologic diseases affecting retinal assessment	Mean 70 ± 8 years	77% female	NR	NR	3 years (36 months)	48 eyes progressed: 40 atrophic AMD, 8 neovascular AMD
Seddon et al. [32]	Derivation cohort (DP): AREDS participants; validation cohort (VC): multiple cohorts (White/Caucasian patients)	DP: AREDS enrollment Nov 1992–Jan 1998; VC: multiple cohorts (recruitment years NR)	DP: AREDS eligibility; intermediate AMD or advanced AMD in one/both eyes; blood specimens for genetic testing	DP: AREDS exclusions; VC: first‐degree relatives excluded (per paper)	DP: Progressors 70.2 (SD 5.2); nonprogressors 68.1 (SD 4.7). VC: Progressors 74.1 (SD 7.5); nonprogressors 73.2 (SD 7.8)	DP 56.55% (1648 females); VC 55.77% (546 females)	DP ≈ 96% Caucasian; VC 100% Caucasian (as reported)	Education proxy	DP 8.8 years; VC 6.2 years	DP 809 progressors; VC 294 progressors
Babenko et al. [33]	AREDS participants (US multicentre cohort; fundus‐image deep learning; arXiv preprint)	AREDS enrollment Nov 1992–Jan 1998 (follow‐up through 2001)	AREDS eligibility; fundus photographs available; genetic specimens available (per paper)	Reversals of progression; images containing text; images where circular boundary not detected	Median 69 (IQR 65–73) at baseline	NR	NR (AREDS predominantly White; specific % depends on analysis subset)	NR	1 year (primary prediction horizon)	NR
Schmidt‐Erfurth et al. [34]	HARBOR trial participants: fellow eyes with intermediate AMD at baseline (conversion to late AMD over follow‐up)	HARBOR trial enrollment 2009–2011 (24‐month follow‐up; completion ≈ 2012)	HARBOR RCT enrollment criteria; fellow eye intermediate AMD with imaging available for modeling	Trial exclusions (e.g., prior ocular surgeries/conditions; systemic exclusions)	Mean 78 ± 8 years	59% female	97% Caucasian	NR	2 years	159 fellow eyes converted to late AMD (as reported in paper)
Seddon et al. [11]	AREDS participants (US multicentre cohort)	AREDS enrollment Nov 1992–Jan 1998 (follow‐up extended beyond the trial phase)	AREDS eligibility; age 55–80; VA 20/32 or better in ≥ 1 eye; ocular criteria; genetic specimens	Lost to follow‐up/long‐term follow‐up unlikely (per paper)	Mean ages reported for progressors/nonprogressors (e.g., 70.2 vs 68.1)	≈ 56% female	≈ 96% Caucasian (AREDS)	NR	Varies by baseline AMD status (mean follow‐up reported separately in the paper)	819 progressors (as reported)
Hallak et al. [35]	HARBOR trial: fellow eyes with non‐neovascular AMD at baseline; secondary analysis cohort	HARBOR trial enrollment 2009–2011 (secondary analysis published later; data collection/analysis dates not recruitment)	HARBOR participants; fellow eye non‐neovascular AMD at baseline; imaging features extracted	NR	Mean 78.12 (SD 8.28)	59.2% female	NR	NR	2 years	154 eyes converted to neovascular AMD (22.4%)
Banerjee et al. [13]	HARBOR trial fellow‐eye dataset plus an external Miami dataset (as described in paper)	HARBOR enrollment 2009–2011; external Miami cohort recruitment years NR	Adults with AMD diagnosis; longitudinal SD‐OCT available; consent and follow‐up per cohort	Other ocular diseases affecting outcomes; recent ocular surgery; systemic diseases affecting follow‐up; inability to comply (as described)	HARBOR mean 78.2; Miami mean 75.3	HARBOR 59.6%; Miami 63.6%	HARBOR: 96.9% White; 1.6% Asian; 0.4% Black; 0.3% American/Alaska Native; 0.3% Native Hawaiian/Pacific Islander	NR	2 years	149 progressed out of 671 fellow eyes (as reported)
Bhuiyan et al. [36]	AREDS participants with gradable color fundus photographs	AREDS enrollment 1992–1998	Participants enrolled in AREDS with gradable images and longitudinal follow‐up data	Ungradable images; missing follow‐up or outcome data	Mean ≈ 68.4 years	≈ 56% female	Predominantly White (> 95%, AREDS cohort)	NR	5‐year risk prediction (AREDS longitudinal follow‐up)	Progression to late AMD at 5 years (exact count not emphasized in paper)
Wu et al. [37]	LEAD trial: bilateral large drusen without late AMD (same cohort as [31])	July 26, 2012–April 30, 2015	Bilateral large drusen; no late AMD on multimodal imaging	Late AMD at baseline	Mean 70 ± 8 years	77% female	NR	NR	3 years (36 months)	48 eyes progressed: 40 atrophic AMD, 8 neovascular AMD
Yim et al. [38]	Moorfields Eye Hospital NHS Foundation Trust cohort (OCT; fellow‐eye conversion prediction)	June 2012–June 2017	Unilateral exudative AMD; fellow eye at risk; OCT imaging available	NR	Mean 78.8 (SD 7.6)	62% female	White 71.5%; Asian 23.5%; Black 2.0%; mixed 1.0%; other/unspecified 2.1%	NR	24 months (≈ 2.0 years)	NR
Rivail et al. [39]	Intermediate AMD cohorts evaluated for progression to atrophy; external validation used the LEAD cohort (see Wu et al. [31])	NR	Intermediate AMD with longitudinal OCT imaging available	NR	NR	NR	NR	NR	Longitudinal OCT follow‐up; duration NR (time‐to‐event analysis)	Atrophy events reported separately for internal and external cohorts
Russakoff et al. [40]	71 eyes of 71 patients with early/intermediate AMD and contralateral wet AMD; imaged with SD‐OCT at baseline, year 1, and year 2 (Moorfields/UCL, UK)	NR (longitudinal imaging over 2 years: baseline, year 1, year 2)	Confirmed early/intermediate AMD in study eye with contralateral wet AMD; SD‐OCT imaging available at baseline and follow‐up	Geographic atrophy (GA) cases excluded (focus on CNV/wet AMD conversion)	Mean 74 (SD 8.5); median 76; range 57–91	60.6% female (43/71)	NR	NR	≈ 2.0 years (mean interval baseline⟶year 2: 2 years; SD 2 months)	31/71 converted to wet AMD by year 2
Burlina et al. [41]	AREDS participants with baseline fundus photographs and known 5‐year outcomes	AREDS enrollment 1992–1998	Gradable baseline fundus images and documented 5‐year AMD status	Ungradable images; missing outcome data	Mean ≈ 68.4 years	≈ 56% female	Predominantly White (> 95%)	NR	5 years	454 progressed to late AMD within 5 years
de Sisternes et al. [42]	Patients with early/intermediate nonexudative AMD	Retrospective 5‐year SD‐OCT dataset	Early/intermediate AMD at baseline + ≥ 1 follow‐up visit	No additional exclusions reported	Mean age 80.98 (SD 8.05) years	72.03% female (overall cohort)	Not reported	Not reported	Mean ≈ 6.16 months between visits (≈0.5 yr intervals)	36 eyes progressed to exudative AMD
Seddon et al. [43]	AREDS participants (risk prediction for advanced AMD)	AREDS enrollment Nov 1992–Jan 1998 (follow‐up extended beyond the trial phase)	AREDS participants aged 55+ with graded AMD severity and covariates/genetics	NR	Progressors mean ≈ 70.2; nonprogressors mean ≈ 68.1 (as reported)	≈ 55%–57% female	NR	NR	NR	834 progressors to advanced AMD (as reported)
Sitnilska et al. [15]	Early or intermediate AMD without late AMD at baseline	Baseline: June 2008–Jan 2012; follow‐up: Apr 2014–Nov 2016	Early/intermediate AMD; no CNV or GA; ≥ 5 years follow‐up	Other retinal pathology; high myopia ≥ 6D; severe macular pucker; poor‐quality imaging	70.1 ± 6.2 years	56.9%	NR	NR	5.94 ± 0.91	52/232 (22.4%)
Ajana et al. [44]	Adults ≥ 55 years (RS‐I); ≥ 73 years (ALIENOR) without advanced AMD at baseline	RS‐I: 1990–1993 baseline; ALIENOR: 2006–2008 baseline	No advanced AMD at baseline; ≥ 1 follow‐up visit; complete phenotypic, genetic, lifestyle data	Missing genetic data; missing phenotypic data; missing lifestyle data	RS‐I: ≥ 55 yrs; ALIENOR: ≥ 73 yrs	Not specified overall (reported within cohorts)	European (Netherlands and France cohorts)	Education included as predictor (primary/secondary/higher)	RS‐I: 10.8 years; ALIENOR: 6.5 years (median)	RS‐I: 108; ALIENOR: 33

Abbreviations: AMD, age‐related macular degeneration; AREDS, Age‐Related Eye Disease Study; BCVA, best‐corrected visual acuity; BDES, Beaver Dam Eye Study; BMES, Blue Mountains Eye Study; CNV, choroidal neovascularization; DP, derivation population; GA, geographic atrophy; LEAD, laser intervention in early stages of age‐related macular degeneration; NR, not reported; OCT, optical coherence tomography; RS‐I, Rotterdam Study I; SD‐OCT, spectral‐domain OCT; SES, socioeconomic status; VA, visual acuity; VC, validation cohort.

Study‐specific outcome terminology was retained during extraction. In the synthesis, late or advanced AMD refers collectively to late‐stage outcomes, whereas geographic atrophy or macular atrophy and neovascular AMD, choroidal neovascularization, or macular neovascularization refer to the atrophic and neovascular subtypes, respectively.

### 2.2. Information Sources and Search Strategy

A comprehensive search strategy was executed across electronic databases, including PubMed/MEDLINE, and Embase, from their inception until 2022‐04‐05. The search was updated on 2024‐12‐12 to include new studies. The updated search applied identical eligibility criteria, screening procedures, and data extraction methods as the original search. Detailed search strategies, including keywords and filters applied, are available in Supporting Appendix A. Additionally, reference lists of included studies were hand‐searched and PubMed and Google Scholar were utilized to identify further relevant articles.

### 2.3. Study Selection

Three independent reviewers (RA, MQ, and SS) screened titles and abstracts. Full‐text articles of potentially relevant studies were independently assessed against predefined inclusion and exclusion criteria. Disagreements were resolved through discussion and consensus, with a fourth reviewer (VC) consulted as needed. The distinction between association (risk‐factor) studies and prediction‐model studies was applied at title/abstract screening and confirmed at full text using an a priori definition: eligible studies combined multiple predictors into an individualized estimate of future risk and reported model performance. Uncertain records were advanced to full‐text review, and reference lists of included studies were hand‐searched, to reduce the risk of omitting eligible model‐development studies. Studies centered on a single novel predictor remained eligible when it was assessed as an addition to a multivariable model with reported predictive performance.

### 2.4. Data Extraction and Data Items

Data extraction was conducted independently by two reviewers (RA and SS) using a standardized form. Extracted information included study design and data source; sample size and population characteristics; outcome definitions; prediction horizon or horizons, defined as the interval between baseline assessment and the predicted outcome; predictor domains; model type and development approach; validation strategy; and reported model performance metrics. When studies reported multiple models or prediction horizons, each was extracted and summarized separately to capture the range of modeling approaches and time horizons evaluated; however, this did not imply equal weighting of models or studies in the synthesis, which was conducted at the study level. Risk of bias was assessed at the study level, consistent with PROBAST guidance for systematic reviews of prediction models.

### 2.5. Model Development Methods

For each included study, model development methods were evaluated, focusing on selection of candidate variables, strategies for handling missing data, and statistical models employed.

### 2.6. Metrics of Model Performance

Model performance metrics were extracted as reported. Discrimination was most commonly reported using the area under the receiver operating characteristic curve (AUC‐ROC) or c‐statistic. Calibration was considered quantitatively assessed only when studies reported calibration plots, calibration slope, calibration‐in‐the‐large, Brier score, or equivalent numerical measures. Studies that referenced calibration without presenting quantitative results were classified as having incomplete calibration reporting, and absence of calibration assessment was treated as a methodological limitation.

Validation strategies were categorized as internal or external. External validation was defined as evaluation in an independent dataset distinct from the development sample. No minimum standard of external validation was imposed.

### 2.7. Risk of Bias Assessment

Risk of bias in included studies was assessed using the PROBAST, designed specifically for evaluating risk of bias in studies developing or validating prediction models. Each study was assessed across four domains: participant selection, predictor measurement, outcome measurement, and statistical analysis. Two reviewers (RA and SS) independently evaluated each included study, documented their assessments, and resolved disagreements through consensus. The PROBAST tool aids in identifying potential biases that may affect the validity of predictive models, providing a comprehensive understanding of the reliability of the findings.

### 2.8. Data Synthesis

In accordance with the prespecified conditional analysis plan, meta‐analysis was not undertaken. Pooling of discrimination metrics was judged inappropriate because it would combine models developed for different outcomes, prediction horizons, baseline‐risk strata, and analytic units, obscuring rather than clarifying performance. Findings were therefore synthesized narratively, with structured comparison of model characteristics, performance reporting, validation approaches, and risk of bias.

## 3. Results

### 3.1. Study Selection

**FIGURE 1:**
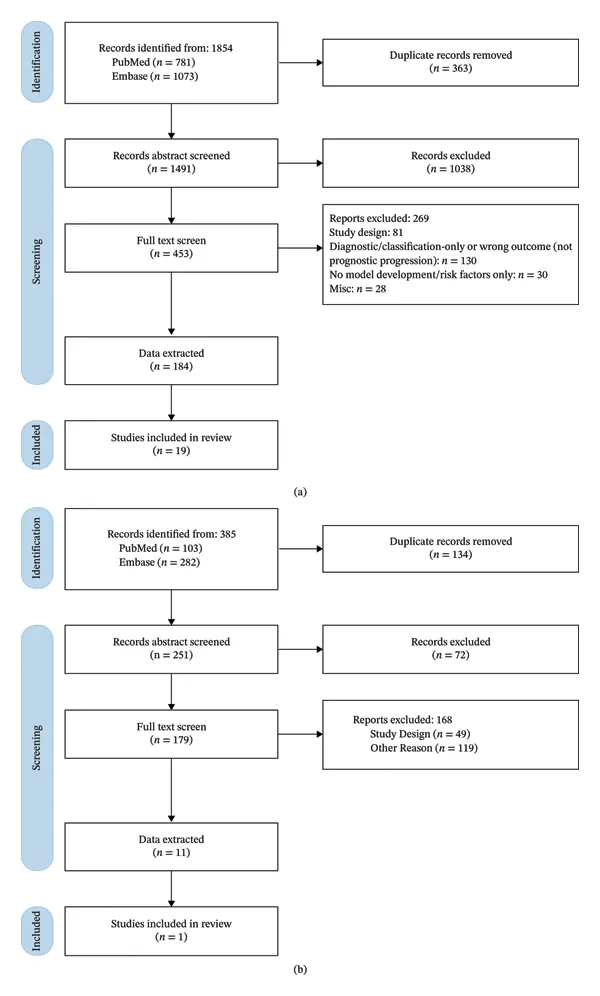
(a) PRISMA flow diagram for the initial search (5 April 2022). (b) PRISMA flow diagram for the updated search (12 December 2024).

The initial database search identified 1854 records and the updated search identified 385 records (total 2239). After removal of 497 duplicate records, 1742 unique titles and abstracts were screened. Of these, 632 reports were assessed for eligibility (453 from the initial search and 179 from the updated search). Following full‐text assessment, 20 studies met all predefined eligibility criteria and were included in the systematic review: 19 from the initial search and one from the updated search. The initial and updated searches followed the same selection process; full‐text exclusion reasons are reported separately for each search in the PRISMA flow diagrams (Figure 1a,b).

### 3.2. Study Characteristics

The twenty included studies [11–13, 15, 29–44] focused on prognostic models for progression from nonlate to late AMD. A summary of key study characteristics can be found in Table 2. Studies drew primarily from the United States and Australia, with additional cohorts from the United Kingdom, the Netherlands, France, Northern Ireland, and Austria; several studies used multicountry datasets.

**TABLE 2:** Summary of study characteristics, model types, and performance metrics for risk prediction in age‐related macular degeneration.

Study ID	Model purpose	Setting/context	Data source	Sample size (*n*)	Predictor (*n*) candidate	Final (*n*)	Prediction horizon (yr)	Outcome to be predicted	Outcome definitions/classifications	Model performance measure
Chiu et al. [12]	Risk	United States (AREDS), Australia (BMES)	AREDS; validation: BMES	AREDS: 8177 eyes at risk (1185 affected; 6992 unaffected); BMES: 2169 participants/3763 eyes at 10‐year follow‐up	8	8	Up to 8 years (AREDS) and 10 years (BMES)	Advanced AMD	Advanced AMD defined as neovascularization or central geographic atrophy; graded from stereoscopic fundus photographs (AREDS protocol; BMES Wisconsin system)	AUC/C‐index; sensitivity; specificity
Buitendijk et al. [29]	Risk	Three Continent AMD Consortium (RS, BDES, BMES)	Rotterdam Study; Beaver Dam Eye Study; Blue Mountains Eye Study	Three population‐based cohorts (RS, BDES, BMES); pooled set *N* = 10,106 at risk of incident late AMD	Genetic, environmental, and baseline AMD phenotype variables (26 SNPs plus demographic and lifestyle factors)	6 (age, sex, 26‐SNP genetic risk, smoking, body mass index, baseline AMD phenotype)	Time‐to‐event over follow‐up (median 11.1 years; 4–5 visits); no fixed horizon	Incident late AMD	AMD graded on fundus photos using 3CC AMD severity scale	AUC (discrimination); risk score with hazard ratios
Yan et al. [30]	Prediction	United States (AREDS)	AREDS	1351 subjects (training 1223; test 128) with usable baseline and follow‐up fundus images	Image input (no fixed prespecified predictors)	N/A (end‐to‐end DL)	2–7 years (multihorizon progression time prediction)	Progression to late AMD/progression time	Fundus images graded by central reading center; late AMD defined by standard AREDS late AMD endpoints	Discrimination (AUC) across horizons
Wu et al. [31]	Risk	Australia and Northern Ireland (LEAD trial; sham arm)	LEAD trial sham‐treatment arm	140 participants (280 eyes)	Up to 9 (age + CFP + OCT biomarkers across specified models)	Model‐dependent (CFP‐only/OCT‐minimal/OCT‐extended/combined)	3 years	Late AMD/atrophic endpoints (trial‐defined)	Modified Wisconsin grading on CFP; late AMD includes nAMD/GA and atrophic changes (nGA/cRORA)	AUC/c‐statistic; *R* ^2^ also reported
Seddon et al. [32]	Risk	Derivation: US (AREDS); validation: clinical cohorts in ongoing studies	AREDS derivation + external validation cohorts	Derivation: 2914 subjects (809 progressors); external validation: 980 subjects (294 progressors)	14	14	5 and 10 years	Progression to geographic atrophy or neovascular AMD	Clinical Age‐Related Maculopathy Staging System (stage progression to late AMD subtypes)	C‐statistic/AUC
Babenko et al. [33]	Prediction	United States (AREDS)	AREDS (arXiv preprint)	4628	Image input (no fixed prespecified predictors)	N/A	1 year	Neovascular AMD	Fundus images graded by central reading center; severity scales used as labels/auxiliary endpoints	AUC; sensitivity; specificity
Schmidt‐Erfurth et al. [34]	Prediction	United States (HARBOR trial context)	HARBOR clinical trial (fellow eyes)	Population and study subsets reported (e.g., ≈1097 population; ≈495 fellow eyes)	41	41	20 months	Advanced AMD (CNV or GA)	OCT images with expert/manual grading; conversion defined by CNV/GA endpoints	AUC
Seddon et al. [11]	Risk	United States (AREDS)	AREDS	2937 participants	17	17	Up to 10 years	Advanced AMD	Development of neovascular AMD or central geographic atrophy	C‐statistic; calibration; reclassification
Hallak et al. [35]	Risk	United States (HARBOR trial)	HARBOR clinical trial	686	Demographic + genetic + imaging biomarkers	NR (final retained predictors reported in multivariable model)	12 months	Conversion to neovascular AMD	Conversion defined by CNV development confirmed via imaging/clinical criteria	C‐statistic/AUC (values NR; results reported as hazard ratios)
Banerjee et al. [13]	Risk	United States (HARBOR) + US clinic dataset (Bascom Palmer)	HARBOR dataset + Bascom Palmer Eye Institute dataset	HARBOR: 674 patients; Bascom Palmer: 834 patients	21	21	3, 6, 9, 12, 15, 18 months	Progression/exudation/new MNV	Progression documented by exudation/MNV events between visits	AUC; concordance index
Bhuiyan et al. [36]	Risk	United States (AREDS)	AREDS fundus images	≈58,000 images from ≈4500 participants	Image‐derived features + demographics (model‐dependent)	N/A (end‐to‐end + derived severity probabilities)	1–2 years (incidence) and longer horizons reported	Progression to late AMD	Late AMD defined by standard AREDS late endpoints	C‐statistics/AUC (values NR); calibration metrics referenced
Wu et al. [37]	Risk	Australia and Northern Ireland (LEAD study)	LEAD study	140 participants (280 eyes)	Functional + imaging predictors	4	3 years	Progression to late AMD	Late AMD progression determined at 6 monthly follow‐up; predictive value of microperimetry and low‐luminance deficit compared with CFP pigmentary abnormalities	C‐statistics/AUC
Yim et al. [38]	Prediction	United Kingdom (Moorfields Eye Hospital)	Moorfields Eye Hospital OCT dataset	Test set: 386 unique patients; 5581 total OCT scans (full dataset larger; NR for training/validation Ns)	OCT volumes + automated tissue maps	N/A	6 months	Imminent conversion to exudative AMD in fellow eye	Ground truth defined by first anti‐VEGF injection within 6 months (and related definitions in paper)	AUC; sensitivity/specificity at operating points
Rivail et al. [39]	Prediction	Austria (Vienna intermediate‐AMD cohort); Australia and Northern Ireland (LEAD external cohort)	Vienna longitudinal OCT cohort; external validation in LEAD	Internal: 231 eyes/121 patients; external: 280 eyes/140 patients	OCT scans (learned representations; no hand‐crafted predictor list)	N/A	6, 12, 18 months	Onset of macular atrophy	Macular atrophy onset identified from longitudinal OCT imaging	Dynamic AUC; concordance index (C‐index)
Russakoff et al. [40]	Prediction	United Kingdom	SD‐OCT imaging dataset	71	OCT‐only DL; plus 32‐dim segmentation biomarker vector + patient info (traditional ML)	N/A	2 years	Conversion to neovascular (wet) AMD	Conversion to wet AMD confirmed on SD‐OCT at year 1 or year 2; geographic atrophy cases excluded	AUC
Burlina et al. [41]	Risk	United States (AREDS)	AREDS fundus images	4613 participants; 67,401 fundus images	Image input (learned features)	N/A	5 years	Progression to advanced AMD	Risk estimated via severity classification linked to AREDS outcomes	Weighted *κ* (severity); mean unsigned error (risk); AUC NR
de Sisternes et al. [42]	Risk	United States (single‐center longitudinal SD‐OCT cohort)	Longitudinal SD‐OCT cohort	244 patients with AMD	Quantitative OCT biomarkers (multiple)	Model‐dependent	6 months to 5 years	Progression to exudative AMD	Progression defined by maintaining early/intermediate vs converting to exudative at follow‐up	AUC (time‐dependent; varies by horizon)
Seddon et al. [43]	Risk	United States (AREDS)	AREDS	2951	Genetic + demographic + environmental + macular covariates (10‐variant genetic model)	10	2, 5, 10 years	Progression to advanced AMD	GA or neovascular AMD in at least one eye	AUC/C‐statistic; reclassification
Sitnilska et al. [15]	Risk	Prospective observational cohort (early/intermediate AMD)	EUGENDA multicentre database (Cologne and Radboud UMC)	232	36 SNPs + demographic + systemic + imaging biomarkers (> 40 total)	5 (Age, CFH rs1061170, Pigment abnormalities, DPED, HRF)	Mean 5.9 (SD 0.9)	Progression to late AMD	Development of CNV and/or geographic atrophy (Beckman classification)	AUC = 0.969 (95% CI 0.948–0.990); Hosmer–Lemeshow *p* = 0.797
Ajana et al. [44]	Risk	Netherlands (Rotterdam Study I) and France (ALIENOR)	Rotterdam Study I; ALIENOR	RS‐I (development): 3838 participants (108 incident advanced AMD); ALIENOR (external validation): 362 participants (33 incident advanced AMD)	Candidate predictors include phenotype + lifestyle + clinical + 49‐SNP GRS	7	5, 10, 15 years	Incident advanced AMD	Incident advanced AMD (atrophic, neovascular, or both)	AUC/C‐statistic (time‐dependent); calibration assessment reported

Abbreviations: 3CC, Three Continent Consortium; AMD, age‐related macular degeneration; AREDS, Age‐Related Eye Disease Study; AUC, area under the curve; BCVA, best‐corrected visual acuity; BDES, Beaver Dam Eye Study; BMES, Blue Mountains Eye Study; CFP, color fundus photography; CI, confidence interval; CNV, choroidal neovascularization; cRORA, complete retinal pigment epithelium and outer retinal atrophy; DP, derivation population; GA, geographic atrophy; GRS, genetic risk score; HRF, hyperreflective foci; iAMD, intermediate AMD; LEAD, laser intervention in early stages of age‐related macular degeneration; MNV, macular neovascularization; nAMD, neovascular AMD; nGA, nascent geographic atrophy; NR, not reported; OCT, optical coherence tomography; RS, Rotterdam Study; SD‐OCT, spectral‐domain OCT; SNP, single‐nucleotide polymorphism; VA, visual acuity; VC, validation cohort.

Evidence was concentrated within a small number of recurring datasets. Eight studies were based on the Age‐Related Eye Disease Study (AREDS), three used the HARBOR clinical trial, and two studies developed models in the LEAD trial, which also served as the external cohort for one further study. Population‐based cohorts included the three continent AMD consortium and the Rotterdam Study, with external validation in ALIENOR. Several studies used clinic‐based longitudinal OCT datasets, most notably from Moorfields Eye Hospital. Population details for each model, including cohort descriptions and recruitment settings, are provided in Table 1 for further reference.

Sample sizes varied widely, ranging from 71 participants in the smallest imaging study to 10,106 individuals at risk in pooled population‐based analyses. Imaging‐based studies often reported image‐level sample sizes, with up to 67,401 fundus photographs or more than 5500 OCT scans. Reporting of outcome event counts was inconsistent, limiting assessment of events per predictor in several studies.

Across studies, participants were predominantly older adults, with mean ages typically in the late 60s to late 70s. Sex distributions were generally balanced or modestly female‐predominant. Reporting of race and ethnicity was inconsistent; when reported, most cohorts were predominantly White, particularly in AREDS‐ and HARBOR‐based analyses. Only one large OCT cohort reported substantial representation of non‐White participants.

### 3.3. Outcome and Prediction Horizons

Most models (16 of 20 studies) predicted progression to late or advanced AMD, typically defined as incident neovascular AMD, geographic atrophy, or both. Four studies focused specifically on short‐term exudative or neovascular conversion, occasionally using initiation of anti‐VEGF therapy as a proxy outcome.

Prediction horizons are interpreted here in relation to baseline disease state and event incidence rather than in isolation. Shorter horizons (3–18 months) were typically paired with imminent exudative conversion in enriched clinic or fellow‐eye cohorts, whereas longer horizons (up to 15 years) addressed incident advanced AMD in population‐based cohorts; neither is inherently preferable, and each answers a different clinical question. Because studies defined progression differently, including composite late AMD, neovascular conversion, geographic atrophy, and initiation of anti‐VEGF therapy, and applied different prediction horizons, case mixes, and analytic units, reported discrimination values are not directly comparable across models.

### 3.4. Model Types and Predictors

Seven studies used traditional regression‐based or survival models, including points‐based risk scores and Cox proportional hazards models. Two studies applied machine‐learning methods to structured, nonimaging data. Most studies (11 of 20) used machine‐learning or deep‐learning approaches applied to retinal imaging, including fundus photography, OCT volumes, or derived imaging biomarkers. Two studies explicitly incorporated longitudinal modeling using sequences of visits or learned longitudinal representations [13, 39].

**FIGURE 2:**
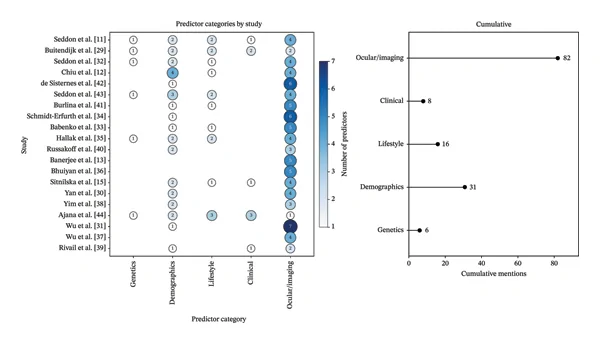
Predictor domains used in the included AMD prognostic models (*n* = 20). Left panel: number of predictors recorded in each domain for each study; circle size and shading indicate the number of predictors. Right panel: cumulative mentions by domain (ocular/imaging 82, demographics 31, lifestyle 16, clinical 8, and genetics 6).

Predictor domains were dominated by ocular and imaging variables. Across studies, ocular or imaging predictors were used far more frequently than demographic, clinical, lifestyle, or genetic predictors. Imaging‐based models often treated the image itself as the primary predictor input, whereas cohort‐based models more commonly incorporated age, baseline AMD severity, smoking status, or genetic risk scores. Although baseline ocular/imaging features and age were the most frequently retained predictors, their independent prognostic contribution was not consistently confirmed in external data; we therefore describe these predictors as recurrent rather than confirmed, reflecting modeling convention and biological plausibility rather than validated incremental value. Refer to Figure 2 for a summary of the predictors used in the included models.

### 3.5. Model Performance

**FIGURE 3:**
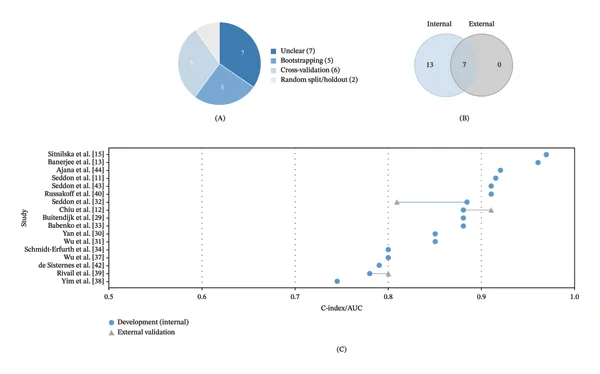
Internal and external validation efforts and discrimination performance across included prediction models. (A) Internal validation methods among all prediction models (*n* = 20). (B) Overlap between internal and external validation; all seven externally validated studies also reported internal validation, so no study falls in the external‐only region. (C) Study‐level distribution of AUC/c‐index values in development and external validation samples. Where a study reported multiple estimates, the value for its primary model and prediction horizon is plotted; the full set of reported estimates appears in Table 3. Of the seven studies reporting external validation, four reported a numeric external estimate. Hallak, Burlina, and Bhuiyan are omitted because no discrimination estimate was reported. Connecting lines indicate the direction of the performance difference: gray where the external estimate is higher and blue where the development estimate is higher.

Discrimination was the most frequently reported performance metric. AUC or c‐statistics ranged from approximately 0.65 to 0.97 across models, with many estimates between 0.80 and 0.92. Several studies reported multiple discrimination estimates across outcomes or prediction horizons. This range spans all eligible reported models, including secondary outcomes and exploratory horizons; when restricted to each study’s primary model and estimate (where declared), discrimination was more tightly clustered (approximately 0.80–0.92), and studies not declaring a primary model are noted as unclear in Table 3. Where reported, 95% confidence intervals or standard errors are given alongside point estimates (Table 3); intervals were not recomputed from sample size alone because valid estimation also requires event counts and, in several studies, adjustment for clustered eyes, repeated scans, or censoring. The highest discrimination estimates (*c*‐statistic > 0.92) occurred in selected clinical or imaging cohorts rather than population‐based cohorts, consistent with narrower case mix. Refer to Table 3 and Figure 3C for model performance data.

**TABLE 3:** Summary of discrimination, calibration, and validation metrics for age‐related macular degeneration prediction models.

Study ID	Model type	Discrimination	Calibration reported	Model fit reported	Reclassification reported	Validation (internal, external)	Overall performance measure
Chiu et al. [12]	Logistic regression	AREDS c‐index = 0.88; BMES c‐index = 0.91; sensitivity = 87.6%; specificity = 73.6% (threshold dependent)	Yes	Yes	No	Internal, yes; external, yes	Brier score = 0.09
Buitendijk et al. [29]	Cox proportional hazards	RS AUC = 0.88; BDES/BMES AUC = 0.85; pooled AUC = 0.87	No	No	No	Internal, yes; external, no	NR
Yan et al. [30]	Deep learning	Image + genotype: Youden accuracy = 78.4% (SD 1.6%); AUC = 0.85 (avg)	Yes	Yes	No	Internal, yes; external, no	NR
Wu et al. [31]	Cox proportional hazards	Late AMD: M1 AUC = 0.80; M2 0.82; M3 0.82; M4 0.85. Atrophic AMD: M1 0.83; M2 0.84; M3 0.85; M4 0.88	No	No	No	Internal, yes; external, No	*R* ^2^ (explained variation)
Seddon et al. [32]	Cox proportional hazards	Model 4 (all‐risk factors): 5y DP 0.858, VC 0.750; 10y DP 0.884, VC 0.809	No	No	Yes	Internal, yes; external, yes	NR
Babenko et al. [33]	Deep learning	None/early/iAMD: Sens 78 ± 6, Spec 80 ± 2; iAMD: Sens 57 ± 6, Spec 81 ± 4; AUCs: 0.88 ± 0.02 and 0.77 ± 0.04	No	No	No	Internal, yes; external, no	NR
Schmidt‐Erfurth et al. [34]	Random forest	Specificity at sensitivity 0.80: CNV 0.46; GA 0.69; AUC: CNV 0.68; GA 0.80	No	No	No	Internal, yes; external, no	NR
Seddon et al. [11]	Cox proportional hazards	C‐statistic: 5y 0.885; 10y 0.915	Yes	No	No	Internal, yes; external, no	NR
Hallak et al. [35]	Cox proportional hazards	NR (no AUC/C‐index reported; results reported as HRs with Kaplan–Meier/log‐rank)	No	Partial	No	Internal, yes; external, no	NR
Banerjee et al. [13]	Deep learning	Deep sequence model: 3 mo AUC = 0.96 ± 0.02; 21 mo AUC = 0.97 ± 0.02. Random forest: 3 mo AUC = 0.65 ± 0.07. Cox: 3 mo mean c‐index = 0.68	No	Yes	Yes	Internal, yes; external, yes	NR
Bhuiyan et al. [36]	Hybrid/ensemble	NR (no discrimination estimate reported for the prognostic model)	No	No	No	Internal, yes; external, yes (NAT‐2)	NR
Wu et al. [37]	Logistic regression	AUC = 0.68 (microperimetry) and 0.80 (CFP grading)	No	No	No	Internal, yes; external, no	NR
Yim et al. [38]	Deep learning	Per‐scan AUC = 0.745 (conversion scan ground truth); AUC = 0.886 (injection‐date ground truth)	No	No	No	Internal, yes; external, no	NR
Rivail et al. [39]	Deep learning	Internal (MUV cohort, cross‐validation): CoxPH dynamic AUC = 0.80 (95% CI 0.749–0.849), C‐index = 0.78 (0.735–0.833); deep survival model comparable. External (LEAD): deep survival model dynamic AUC = 0.82 (0.773–0.867), C‐index = 0.80 (0.745–0.846)	No	No	No	Internal, yes; external, yes (LEAD)	NR
Russakoff et al. [40]	Deep learning	AUC (AMDnet + preprocessing): 0.89 (B‐scan level), 0.91 (volume/patient level). AUC (VGG16+preprocessing): 0.82 (B‐scan), 0.87 (volume). Traditional ML (SVM with 32 segmentation features + age/sex): mean AUC = 0.79.	No	Partial	No	Internal, yes; external, no	NR
Burlina et al. [41]	Deep learning	NR (focus on severity characterization + 5‐year risk estimation; performance includes weighted *κ*/errors)	No	No	No	Internal, yes; external, No	NR
de Sisternes et al. [42]	Logistic regression	AUC by elapsed time: 11 months 0.92 (95% CI 0.83–0.98); 16 months 0.86 (0.77–0.95); 18 months 0.70 (0.54–0.84); 48 months 0.79 (0.69–0.87)	No	No	Yes	Internal, yes; external, no	NR
Seddon et al. [43]	Cox proportional hazards	C‐statistic = 0.91 (genetic + environmental model)	Yes	Yes	Yes	Internal, yes; external, yes	NR
Sitnilska et al. [15]	Logistic regression	AUC = 0.969 (95% CI 0.948–0.990); ROC analysis performed	Yes	Yes	No	Internal, yes; external, no	NR
Ajana et al. [44]	Cox proportional hazards (LASSO survival)	AUC: RS‐I 0.92 (5y), 0.92 (10y), 0.91 (15y); ALIENOR 0.92 (5y)	Yes	Yes	No	Internal, yes; external, yes	NR

*Note:* Calibration reported indicates that calibration was mentioned or attempted; see Methods section for the definition of quantitative calibration. Partial indicates that some model‐fit information was reported, but no complete formal model‐fit assessment was provided. Overall performance refers to measures capturing both discrimination and calibration (for example Brier score or *R* ^2^); discrimination‐only measures are given in the Discrimination column.

Abbreviations: AUC, area under the curve; CFP, color fundus photography; CNV, choroidal neovascularization; DP, derivation population; GA, geographic atrophy; iAMD, intermediate AMD; LEAD, laser intervention in early stages of age‐related macular degeneration; NR, not reported; ROC, receiver operating characteristic; SVM, support vector machine; VC, validation cohort.

Calibration reporting varied across studies (Table 3). Six studies mentioned calibration; however, reporting was heterogeneous and often did not meet our criteria for quantitative calibration (i.e., calibration plots, calibration slope, calibration‐in‐the‐large, Brier score, or equivalent numerical measures). Where specified, evaluation relied on goodness‐of‐fit testing (e.g., Hosmer–Lemeshow *p* = 0.797) [15] or other study‐specific approaches in cohort‐based models [11, 12, 43, 44]. Most studies did not report quantitative calibration metrics and were therefore classified as having no calibration reported in Table 3. Two studies reported an overall performance measure explicitly: a Brier score of 0.09 and an *R* ^2^ statistic for explained variation [12, 31].

### 3.6. Validation

All included studies reported some form of internal model evaluation; however, reporting of validation methods was often incomplete. Internal validation approaches included bootstrapping (5 studies), cross‐validation (6 studies), and random split or holdout validation (2 studies). In seven studies, the internal validation strategy was not described in sufficient detail to classify. External validation was reported in 7 of 20 studies, but its rigor varied. Several studies used cohorts that were related or similarly selected, rather than being clearly distinct in health system, device, or demographic composition; calibration was seldom reported in the external sample; and no study reported recalibration or model updating. The presence of external validation therefore should not be equated with confirmed transportability. Refer to Figure 3A,B for a summary of validation strategies.

### 3.7. Risk of Bias Assessment

**FIGURE 4:**
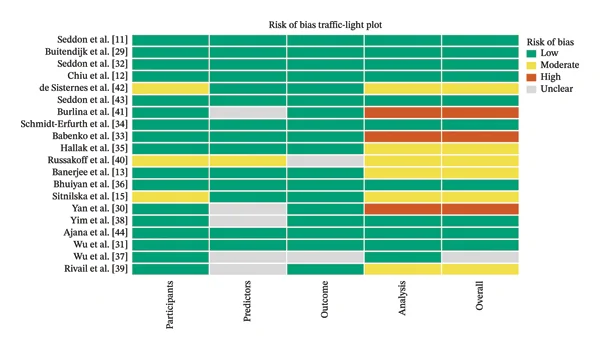
Risk‐of‐bias assessment of included studies using PROBAST domains. Traffic‐light plot showing risk‐of‐bias judgments for each study across four PROBAST domains (participants, predictors, outcome, and analysis) and overall risk of bias. Green indicates low risk, yellow indicates moderate risk, orange indicates high risk, and gray indicates unclear risk.

Risk of bias was assessed using the (PROBAST. Ten of 20 studies were judged to be at low overall risk of bias, six at moderate risk, three at high risk, and one at unclear risk (Figure 4). Thus, although a substantial subset was methodologically sound at the study level, evidence‐level limitations—including infrequent calibration, uncommon external validation, limited population diversity, and heterogeneous outcomes—constrain the body of evidence. Concerns were concentrated in the analysis domain. High or moderate risk ratings in this domain most frequently reflected limited events relative to the number of candidate predictors or model parameters, incomplete reporting of model development procedures, and insufficient description of internal validation strategies. Several studies did not report approaches used to address missing data or relied on complete case analysis without justification. Evaluation of model performance commonly emphasized discrimination, while reporting of calibration or other measures of overall model performance was limited.

Risk of bias in the participants and outcome domains was generally low across studies. Moderate concern in the participants domain occurred when cohort selection procedures or eligibility criteria were incompletely described. Moderate ratings in the outcome domain arose in studies where outcome ascertainment relied on image grading without explicit reporting of masking or standardized grading procedures. Further details on the risk of bias in each domain for each study are provided in Figure 4.

## 4. Discussion

This systematic review provides an appraisal of multivariable prognostic models developed to predict progression from nonlate to late AMD. Unlike earlier reviews centered on diagnostic classification or image‐based disease detection [21, 22, 24, 25], this review evaluates models intended to estimate future risk over a defined time horizon and assesses them against established standards for prediction model development and validation. That distinction matters. Diagnostic models identify existing disease. Prognostic models aim to generate credible estimates of future risk that can support follow‐up, counseling, and risk stratification [9, 10].

Across twenty studies, the literature shows clear technical development but limited methodological maturity. Most evidence is derived from a small number of recurring cohorts, especially AREDS and HARBOR, with additional contributions from LEAD, Moorfields‐based OCT cohorts, and a small number of European population studies [11–13, 34, 35, 43, 44]. This area of research therefore appears broader in publication volume than in underlying population diversity. Many datasets were predominantly White, often drawn from trial populations or clinical settings. That concentration narrows the range of clinical, demographic, and imaging contexts in which these models have actually been tested and limits confidence in transportability to more diverse populations [45].

A homogeneous development cohort is not inherently a flaw: a model developed in a well‐characterized local population can be appropriate for a defined use context, provided the intended use, target population, and limits of transportability are stated explicitly. The concern is not the absence of a worldwide study, but the absence of a clear specification of where a model is expected to perform and evidence that it does so. In practice, however, most models were developed in trials, specialty cohorts, or fellow‐eye populations enriched for progression risk. Such settings offer standardized imaging, tighter eligibility criteria, and more structured follow‐up, all of which can inflate apparent performance [46]; they are also less representative of routine care, and models developed under these conditions may not retain the same performance in broader ophthalmic practice or mixed‐risk populations [47].

Predictor selections were similar across different modeling types. Ocular phenotype and imaging markers dominated, whether models used regression, survival analysis, random forests, or deep learning. Baseline AMD severity, drusen burden, pigmentary abnormalities, age, smoking, and selected genetic variants appeared frequently across a number of studies [11, 12, 15, 29]. Fundus photography and OCT were central in many newer models, either as direct inputs to end‐to‐end architectures or through engineered biomarkers [30, 34, 38, 40]. This suggests that, despite technical heterogeneity, most models are drawing on a common biological core of disease progression. It also raises a validity question: in the case of a few image‐based studies, it is unclear whether the model is learning novel prognostic information or simply automating baseline severity assessment, because the dominant predictive features are closely aligned with established markers of current AMD stage, which are already strong determinants of future progression. [36, 41]. Because diagnostic studies estimate current disease status rather than future risk, their covariates were outside the prespecified scope and were not extracted. Some diagnostic imaging features are nonetheless plausible prognostic candidates; they should be evaluated within prospective prognostic designs that demonstrate temporal association and incremental predictive value before being adopted as prognostic predictors.

The literature is also highly heterogeneous in what it defines as progression. Most studies targeted late AMD, usually neovascular AMD, geographic atrophy, or a composite of the two. Others focused on exudative conversion, fellow‐eye progression, or treatment initiation within a defined period [13, 38]. Prediction horizons ranged from a few months to more than a decade. These are not interchangeable prediction tasks [48]. A model designed to predict 6‐month exudative conversion in a retina clinic cohort is answering a different clinical question from a model estimating 10‐year incident advanced AMD in a population‐based cohort. Studies also used inconsistent analytic units. Some studies modeled eyes, other patients, and others serial scans. These choices influence event rates, sample size, dependence structure, and apparent performance. Eye‐level analyses can be informative, but only if within‐person correlation and repeated measures are handled appropriately and most studies did not report that level of detail to distinguish this. Reported AUC or c‐statistic values therefore cannot be interpreted in isolation from the outcome definition, time horizon, case mix, and analytic unit. This heterogeneity is the reason why a narrative synthesis was more appropriate than quantitative pooling of the identified models.

Conventional models remain highly prevalent and competitive in performance. Cox and logistic models based on clinical phenotype, smoking, demographic factors, and genetic risk frequently achieved discrimination comparable to more complex machine learning approaches, particularly for medium‐ and long‐term prediction [11, 12, 29, 43, 44]. Some recent papers have implied that deep learning has displaced traditional prognostic models [49, 50]; the evidence in this review does not support that claim. Image‐based models have expanded the types of data available for prediction, especially in CFP‐ and OCT‐rich settings, and may be particularly useful for short‐horizon or subtype‐specific tasks [13, 30, 38]. Yet conventional models retain important advantages in transparency, parsimony, and ease of recalibration. For clinical implementation, a slightly less discriminating but well‐calibrated and interpretable model may be more useful than a complex system that performs well only under development conditions.

Most studies reported moderate to strong discrimination, with AUC or c‐statistic values commonly ranging from about 0.80 to 0.92 and extending from 0.65 to 0.97 across the included studies. That pattern indicates that progression to late AMD is prognostically tractable. It does not establish clinical readiness. A model can separate higher‐risk from lower‐risk patients yet still provide inaccurate absolute risk estimates. In prognostic settings, that distinction is essential. Decisions about surveillance intensity, referral, or trial eligibility depend on calibrated risk, not discrimination alone [16, 51]. Only a minority of studies reported quantitative calibration, and reclassification or broader measures of overall performance were rare [11, 12, 15, 43, 44]. The literature has therefore been more consistent at demonstrating signal detection than at establishing reliable absolute risk estimation.

Validation practices reinforce that concern. Internal evaluation was reported in all studies, but methods were often described too briefly to assess rigor with confidence. External validation was uncommon and limited to a small subset of models that reported their external validation results [12, 32, 39, 44]. The scarcity of external validation likely reflects practical constraints, including limited data access and sharing agreements, incompatible imaging devices, inconsistent outcome definitions, and small event counts, rather than investigator neglect. Where fully independent external data are unavailable, temporal validation, multisite collaboration, and federated analysis are feasible alternatives, and the reasons external validation was not performed should be reported transparently. Even when external validation was performed, it often involved related cohorts or similarly selected populations rather than clearly distinct health systems, devices, or demographic settings. This suggests that, within the AMD modeling literature, advances in model development have outpaced progress in model confirmation.

That matters because performance, especially calibration, can degrade when baseline risk, imaging practice, referral thresholds, or patient mix change across settings [17]. Among the few models with both development and external estimates, discrimination was lower in one study [32]; the number of comparable estimates is too small to infer a general direction of change, and this is shown descriptively in Figure 3C.

Risk of bias concerns were clustered in the analysis domain. Common issues included incomplete handling of missing data, limited reporting of events per predictor, unclear control of overfitting, and narrow performance reporting centered on discrimination. These are not peripheral reporting defects. They affect whether published performance estimates are likely to be optimistic. This is especially relevant in studies using high‐dimensional imaging inputs, small samples, enriched event rates, or selected clinic populations, where very strong discrimination may partly reflect restricted case mix or insufficient separation between development and evaluation [15, 34, 40]. The main problem in this literature is not lack of innovation. It is inconsistent adherence to core prognostic modeling principles.

### 4.1. Implications for Future Research

We propose a phased, resource‐conscious pathway rather than a single definitive study: (i) specify target population, outcome, horizon, and decision context; (ii) develop in an existing representative or linked multisite cohort where feasible; (iii) prespecify predictors and size the study by expected events and model complexity; (iv) handle missing data, clustering, and overfitting with robust internal validation; (v) report discrimination, calibration, uncertainty, and clinical utility; (vi) undertake temporal or geographic validation when fully independent data are unavailable; (vii) recalibrate or update before wider use; and (viii) evaluate transportability across sites, devices, and demographic groups before implementation [45]. This is especially critical for genetic and multimodal models, where ancestry, environmental exposures, and access to care can shift predictor distributions and baseline risk [52]. External validation should be routine. At minimum, studies should report calibration‐in‐the‐large and calibration slope. When models are positioned for clinical use, discrimination alone is inadequate. Authors should present decision‐curve analysis or other utility‐based evaluation [53].

Outcome definitions require standardization. Progression should be prespecified, with separate reporting for neovascular AMD, geographic atrophy, and composite late AMD when relevant. Fixed‐horizon prediction and time‐to‐event modeling address different estimates and should be reported as distinct tasks. The same applies to short‐term conversion, subtype‐specific prediction, and long‐term incidence, which should not be collapsed into a single “progression” outcome. Model development also needs stricter analytic discipline: prespecify candidate predictors, use robust internal validation, handle missing data transparently, and prevent leakage across eyes, visits, and patient‐level splits. For longitudinal eye‐based datasets, investigators should state how inter‐eye dependence and repeated measures were handled [54]. These are basic conditions for credible, interpretable, and clinically usable risk estimates.

Imaging‐based prediction remains promising, but the evidentiary standard needs to rise [55]. Models trained on a single camera system, OCT vendor, or grading pipeline may fail when deployed elsewhere. Cross‐device and cross‐site validation should therefore become the standard [56]. Multimodal models also require more disciplined evaluation. The key question is not whether more data can be added, but whether added complexity produces reproducible improvement over strong baseline predictors such as drusen characteristics, age, and smoking.

## 5. Conclusions

Current prognostic models for progression in AMD remain methodologically immature for clinical use. Strong discrimination is reported frequently, but calibration and independent external validation are uncommon, which prevents reliable estimation of absolute risk. The evidence base is further constrained by heavy reliance on a small number of recurring cohorts, limited population diversity, and substantial heterogeneity in outcomes and prediction horizons. Together, these features restrict transportability and increase the risk of miscalibration in new settings. Prognostic modeling in AMD should move beyond discrimination‐centered development toward prespecified horizons, diverse and representative cohorts, rigorous external validation, and routine calibration‐focused evaluation. Until those conditions are met, existing models should be regarded as exploratory rather than clinically actionable tools.

## Author Contributions

R.A. conceptualized and designed the study and wrote the manuscript. P.R. and L.E.G. contributed to the conceptualization, design, and formatting of the manuscript. R.A. and S.S. performed the abstract screening and data extraction. R.A. conducted the analysis, with assistance from P.R. and L.E.G. V.C., P.R., L.E.G., and M.P. provided editorial input and revisions to the manuscript.

## Funding

Lauren E. Griffith was supported by the McLaughlin Foundation Professorship in Population and Public Health. Parminder Raina was supported by the Raymond and Margaret Labarge Chair in Research and Knowledge Application for Optimal Aging. Rumaisa Aljied was supported through doctoral funding provided by Parminder Raina’s endowed chair and Lauren E. Griffith’s grant from the Canadian Institutes of Health Research (CIHR).

## Disclosure

All authors reviewed and approved the final version of the manuscript.

## Ethics Statement

The authors have nothing to report.

## Consent

The authors have nothing to report.

## Conflicts of Interest

The authors declare no conflicts of interest.

## Supporting Information

Additional supporting information can be found online in the Supporting Information section.

## Supporting information


**Supporting Information** The following supporting information is available with the online version of this article. *Supporting Appendix A.* Search strategy: full electronic search strategies as executed, presented line by line for MEDLINE/PubMed and Embase. For each database, the appendix lists the individual concept sets—AMD; prognosis and risk and disease progression; statistical, machine‐learning, and artificial‐intelligence modeling; model performance and validation; predictor domains; and genetic predisposition—together with the Boolean operators used to combine them and the human and English‐language limits applied.

## Data Availability

Data sharing is not applicable to this article, as no new data were created in this study. All data supporting the findings are available from the original publications cited in the reference list.
