# Supplementary material for: Multivariable Prognostic Models for Age‐Related Macular Degeneration: A Systematic Review
Source: J Ophthalmol. 2026 Sep 18;2026:9231744. doi: 10.1155/joph/9231744 (PMC13589163; doi:10.1155/joph/9231744)
Supplement: Supplementary file 1 [file JOPH-2026-9231744-s001.docx]

# Supplementary: Appendix A Search Strategy

This appendix presents the database-specific search strategies as executed. Line numbers correspond to individual concept sets and their combinations (OR/AND), followed by any limits applied.

## Database search strategies

### MEDLINE / PubMed

**MEDLINE / PubMed strategy (as run):**

| **Line** | **Search** |
| --- | --- |
| 1 | "Macular Degeneration"[MeSH] |
| 2 | "age-related macular degeneration"[tiab] |
| 3 | AMD[tiab] |
| 4 | "age-related maculopathy"[tiab] |
| 5 | "macular degeneration"[tiab] |
| 6 | 1 OR 2 OR 3 OR 4 OR 5 |
| 7 | "Prognosis"[MeSH] |
| 8 | "Risk Assessment"[MeSH] |
| 9 | "Risk Factors"[MeSH] |
| 10 | "Disease Progression"[MeSH] |
| 11 | prognos*[tiab] |
| 12 | predict*[tiab] |
| 13 | "risk model*"[tiab] |
| 14 | "prediction model*"[tiab] |
| 15 | "prognostic model*"[tiab] |
| 16 | ((prognos* OR predict*) AND model*)[tiab] |
| 17 | 7 OR 8 OR 9 OR 10 OR 11 OR 12 OR 13 OR 14 OR 15 OR 16 |
| 18 | "Models, Statistical"[MeSH] |
| 19 | "Machine Learning"[MeSH] |
| 20 | "Artificial Intelligence"[MeSH] |
| 21 | "deep learning"[tiab] |
| 22 | "machine learning"[tiab] |
| 23 | "artificial intelligence"[tiab] |
| 24 | "supervised learning"[tiab] |
| 25 | "unsupervised learning"[tiab] |
| 26 | algorithm*[tiab] |
| 27 | "data mining"[tiab] |
| 28 | "predictive analytics"[tiab] |
| 29 | 18 OR 19 OR 20 OR 21 OR 22 OR 23 OR 24 OR 25 OR 26 OR 27 OR 28 |
| 30 | "Validation Studies as Topic"[MeSH] |
| 31 | "model evaluation"[tiab] |
| 32 | "model performance"[tiab] |
| 33 | "predictive accuracy"[tiab] |
| 34 | sensitivity[tiab] |
| 35 | specificity[tiab] |
| 36 | "receiver operating characteristic"[tiab] |
| 37 | "area under curve"[tiab] |
| 38 | discrimination[tiab] |
| 39 | calibration[tiab] |
| 40 | reclassification[tiab] |
| 41 | "external validation"[tiab] |
| 42 | "internal validation"[tiab] |
| 43 | 30 OR 31 OR 32 OR 33 OR 34 OR 35 OR 36 OR 37 OR 38 OR 39 OR 40 OR 41 OR 42 |
| 44 | predictors[tiab] |
| 45 | covariates[tiab] |
| 46 | "clinical features"[tiab] |
| 47 | biomarkers[tiab] |
| 48 | "genetic factors"[tiab] |
| 49 | "environmental factors"[tiab] |
| 50 | "lifestyle factors"[tiab] |
| 51 | "demographic factors"[tiab] |
| 52 | 44 OR 45 OR 46 OR 47 OR 48 OR 49 OR 50 OR 51 |
| 53 | "Genetic Predisposition to Disease"[MeSH] |
| 54 | "Genome-Wide Association Study"[MeSH] |
| 55 | "Genetic Variation"[MeSH] |
| 56 | "Genetics"[MeSH] |
| 57 | "Polymorphism, Genetic"[MeSH] |
| 58 | "Genetic Markers"[MeSH] |
| 59 | "Genetic Testing"[MeSH] |
| 60 | "Genotyping Techniques"[MeSH] |
| 61 | 53 OR 54 OR 55 OR 56 OR 57 OR 58 OR 59 OR 60 |
| 62 | 17 OR 29 OR 43 OR 52 OR 61 |
| 63 | 6 AND 62 |
| 64 | Humans[MeSH] |
| 65 | English[lang] |
| 66 | 63 AND 64 AND 65 |

### Embase.

**Embase strategy (as run):**

| **Line** | **Search** |
| --- | --- |
| 1 | 'age related macular degeneration'/exp |
| 2 | 'macular degeneration'/exp |
| 3 | 'age related macular degeneration':ti,ab |
| 4 | amd:ti,ab |
| 5 | 'age related maculopathy':ti,ab |
| 6 | 'macular degeneration':ti,ab |
| 7 | 1 OR 2 OR 3 OR 4 OR 5 OR 6 |
| 8 | 'prognosis'/exp |
| 9 | 'risk assessment'/exp |
| 10 | 'risk factor'/exp |
| 11 | 'disease progression'/exp |
| 12 | prognos*:ti,ab |
| 13 | predict*:ti,ab |
| 14 | 'risk model*':ti,ab |
| 15 | 'prediction model*':ti,ab |
| 16 | 'prognostic model*':ti,ab |
| 17 | ((prognos* OR predict*) NEAR/3 model*):ti,ab |
| 18 | 8 OR 9 OR 10 OR 11 OR 12 OR 13 OR 14 OR 15 OR 16 OR 17 |
| 19 | 'statistical model'/exp |
| 20 | 'machine learning'/exp |
| 21 | 'artificial intelligence'/exp |
| 22 | 'deep learning':ti,ab |
| 23 | 'machine learning':ti,ab |
| 24 | 'artificial intelligence':ti,ab |
| 25 | 'supervised learning':ti,ab |
| 26 | 'unsupervised learning':ti,ab |
| 27 | algorithm*:ti,ab |
| 28 | 'data mining':ti,ab |
| 29 | 'predictive analytics':ti,ab |
| 30 | 19 OR 20 OR 21 OR 22 OR 23 OR 24 OR 25 OR 26 OR 27 OR 28 OR 29 |
| 31 | 'validation study'/exp |
| 32 | 'model evaluation':ti,ab |
| 33 | 'model performance':ti,ab |
| 34 | 'predictive accuracy':ti,ab |
| 35 | sensitivity:ti,ab |
| 36 | specificity:ti,ab |
| 37 | 'receiver operating characteristic':ti,ab |
| 38 | 'area under curve':ti,ab |
| 39 | discrimination:ti,ab |
| 40 | calibration:ti,ab |
| 41 | reclassification:ti,ab |
| 42 | 'external validation':ti,ab |
| 43 | 'internal validation':ti,ab |
| 44 | 31 OR 32 OR 33 OR 34 OR 35 OR 36 OR 37 OR 38 OR 39 OR 40 OR 41 OR 42 OR 43 |
| 45 | predictors:ti,ab |
| 46 | covariates:ti,ab |
| 47 | 'clinical features':ti,ab |
| 48 | biomarkers:ti,ab |
| 49 | 'genetic factors':ti,ab |
| 50 | 'environmental factors':ti,ab |
| 51 | 'lifestyle factors':ti,ab |
| 52 | 'demographic factors':ti,ab |
| 53 | 45 OR 46 OR 47 OR 48 OR 49 OR 50 OR 51 OR 52 |
| 54 | 'genetic predisposition'/exp |
| 55 | 'genome wide association study'/exp |
| 56 | 'genetic variation'/exp |
| 57 | 'genetics'/exp |
| 58 | 'genetic polymorphism'/exp |
| 59 | 'genetic marker'/exp |
| 60 | 'genetic testing'/exp |
| 61 | 'genotyping'/exp |
| 62 | 54 OR 55 OR 56 OR 57 OR 58 OR 59 OR 60 OR 61 |
| 63 | 18 OR 30 OR 44 OR 53 OR 62 |
| 64 | 7 AND 63 |
| 65 | [humans]/lim |
| 66 | [english]/lim |
| 67 | 64 AND 65 AND 66 |
